# Understanding the Experience of Cancer Pain From the Perspective of Patients and Family Caregivers to Inform Design of an In-Home Smart Health System: Multimethod Approach

**DOI:** 10.2196/20836

**Published:** 2020-08-26

**Authors:** Virginia LeBaron, Rachel Bennett, Ridwan Alam, Leslie Blackhall, Kate Gordon, James Hayes, Nutta Homdee, Randy Jones, Yudel Martinez, Emmanuel Ogunjirin, Tanya Thomas, John Lach

**Affiliations:** 1 University of Virginia School of Nursing Charlottesville, VA United States; 2 University of Virginia School of Engineering & Applied Science Charlottesville, VA United States; 3 University of Virginia School of Medicine Charlottesville, VA United States; 4 Virginia Commonwealth University Health Richmond, VA United States; 5 The George Washington University School of Engineering & Applied Science Washington, DC United States

**Keywords:** cancer, pain, sensors, smart health, caregiver, home based, palliative care, opioids, smart watch

## Abstract

**Background:**

Inadequately managed pain is a serious problem for patients with cancer and those who care for them. Smart health systems can help with remote symptom monitoring and management, but they must be designed with meaningful end-user input.

**Objective:**

This study aims to understand the experience of managing cancer pain at home from the perspective of both patients and family caregivers to inform design of the Behavioral and Environmental Sensing and Intervention for Cancer (BESI-C) smart health system.

**Methods:**

This was a descriptive pilot study using a multimethod approach. Dyads of patients with cancer and difficult pain and their primary family caregivers were recruited from an outpatient oncology clinic. The participant interviews consisted of (1) open-ended questions to explore the overall experience of cancer pain at home, (2) ranking of variables on a Likert-type scale (0, no impact; 5, most impact) that may influence cancer pain at home, and (3) feedback regarding BESI-C system prototypes. Qualitative data were analyzed using a descriptive approach to identity patterns and key themes. Quantitative data were analyzed using SPSS; basic descriptive statistics and independent sample *t* tests were run.

**Results:**

Our sample (n=22; 10 patient-caregiver dyads and 2 patients) uniformly described the experience of managing cancer pain at home as stressful and difficult. Key themes included (1) unpredictability of pain episodes; (2) impact of pain on daily life, especially the negative impact on sleep, activity, and social interactions; and (3) concerns regarding medications. Overall, taking pain medication was rated as the category with the highest impact on a patient’s pain (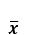
=4.79), followed by the categories of wellness (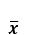
=3.60; sleep quality and quantity, physical activity, mood and oral intake) and interaction (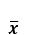
=2.69; busyness of home, social or interpersonal interactions, physical closeness or proximity to others, and emotional closeness and connection to others). The category related to environmental factors (temperature, humidity, noise, and light) was rated with the lowest overall impact (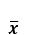
=2.51). Patients and family caregivers expressed receptivity to the concept of BESI-C and reported a preference for using a wearable sensor (smart watch) to capture data related to the abrupt onset of difficult cancer pain.

**Conclusions:**

Smart health systems to support cancer pain management should (1) account for the experience of both the patient and the caregiver, (2) prioritize passive monitoring of physiological and environmental variables to reduce burden, and (3) include functionality that can monitor and track medication intake and efficacy; wellness variables, such as sleep quality and quantity, physical activity, mood, and oral intake; and levels of social interaction and engagement. Systems must consider privacy and data sharing concerns and incorporate feasible strategies to capture and characterize rapid-onset symptoms.

## Introduction

Inadequately managed pain continues to be a serious problem for patients with cancer and those who help care for them. An estimated 40% to 90% of patients with cancer experience pain across the illness continuum [1-3], negatively affecting sleep, adherence to treatment, mood, and overall quality of life [2,4]. Even patients with cancer enrolled in home hospice programs, which are uniquely designed to provide comprehensive support at the end of life, risk experiencing poorly managed symptoms [5-7]. One study found that >50% of hospice patients experience moderate to severe pain in the last week of life [8]. The majority of cancer symptom management occurs in the home setting, where family caregivers commonly play a key role in supporting patients. However, family caregivers are often required to make decisions about symptom management with limited information and support, which can significantly increase emotional distress [4,9,10]. In fact, working to control difficult pain is consistently rated as one of the most stressful tasks performed by family caregivers [11-15].

Ensuring quality home-based symptom management support is especially relevant for patients with advanced disease who may wish to forego aggressive curative treatments, avoid trips to the emergency department and hospitalizations, and focus on comfort care at home. For example, pain that escalates without adequate, prompt treatment can cause significant patient and caregiver distress as well as unplanned health care utilization/emergency department visits, which may not be compatible with patient goals at the end of life [16-19]. Recent studies have estimated that between 25% and 55% of emergency department visits for patients with advanced cancer are avoidable [16,17,20], and uncontrolled pain at home is a major reason that patients disenroll from hospice programs [21-23]. As health care adapts to the challenges and realities of COVID-19, home-based monitoring strategies are likely to become even more essential for seriously ill and immunocompromised patients who will be at higher risk for adverse outcomes if they must present to acute care settings for symptom management.

Although the literature richly describes the experience and consequences of poorly managed cancer pain within the home setting [4,5,24,25], gaps exist in understanding real-time, dynamic contextual factors that may worsen or mitigate the experience of cancer pain from the perspectives of patients *and* family caregivers [4,15,26-30]. Smart health (eg, wireless/mobile technology and user interfaces) is increasingly being utilized to improve remote symptom monitoring and management [31,32], but it is not always designed with meaningful end-user input [33] and may not be appropriate or feasible for the unique needs of patients and caregivers coping with the stressors of advanced, late-stage illness, limiting its ultimate utility and effectiveness [34,35]. Relatedly, ever-evolving technological capabilities can capture a large range of data, but it is not always clear which variables, especially environmental, are most essential and how they should be prioritized [30].

**Figure 1:**
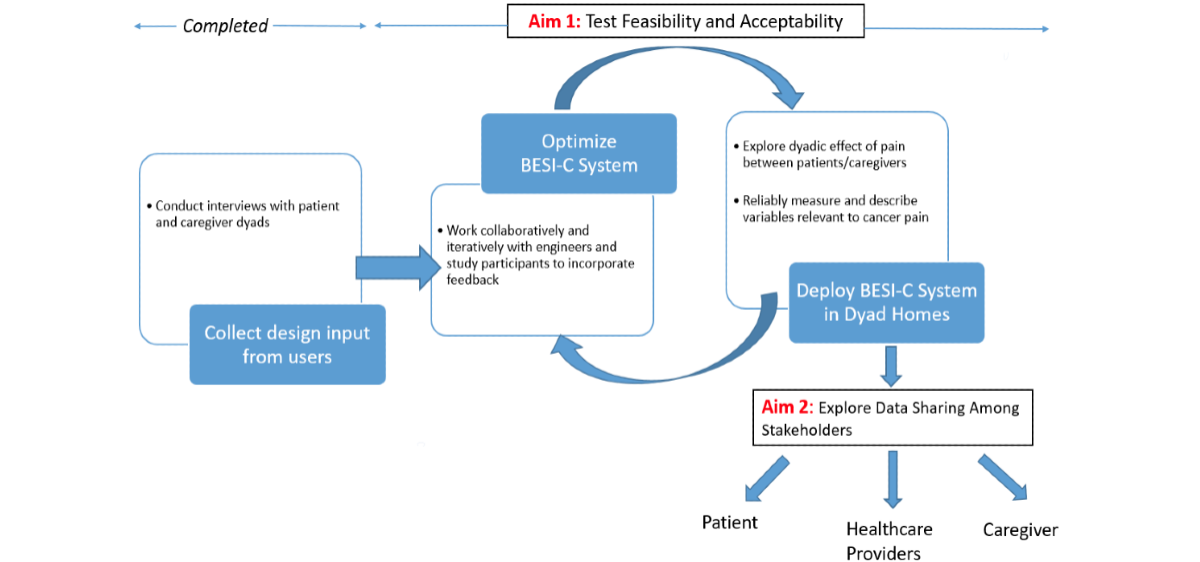
Overall study design. BESI-C: Behavioral and Environmental Sensing and Intervention for Cancer.

This research represents a multiphase effort to design and pilot test an in-home smart health system, known as the Behavioral and Environmental Sensing and Intervention for Cancer (BESI-C) system, with a palliative care oncology population to support patients and family caregivers in monitoring and managing cancer pain. The overall research protocol is described in detail elsewhere [36], but, briefly, BESI-C includes a package of environmental and wearable sensors and user interfaces deployed in patient homes to gather physiological, behavioral, contextual, and environmental data regarding pain events from the perspective of both patients and family caregivers. The ultimate goal of BESI-C is to successfully predict pain episodes and deliver real-time tailored interventions to both patients and caregivers as well as share relevant data with stakeholders. This manuscript presents results from phase I of the project (Figure 1), which aimed, from the perspective of both patients and family caregivers, to (1) explore the general experience and challenges of managing cancer pain in the home setting, (2) evaluate the role of specific variables that may influence cancer pain in the home setting, and (3) gather end-user input to inform BESI-C system design.

## Methods

### Study Design

This was a descriptive pilot study using a multimethod approach.

### Setting

Patients and family caregivers were recruited from an academic palliative care outpatient oncology clinic from April to July 2018.

### Sample

Our goal was to recruit patients and family caregivers managing difficult cancer-related pain in the home setting. Therefore, we used a purposive sampling technique [37], and patient inclusion criteria included (1) diagnosis of locally advanced or metastatic malignancy, (2) currently taking prescribed opioid medications (eg, morphine-type medications) for cancer-related pain, (3) a score of 6 on the National Institutes of Health Patient-Reported Outcomes Measurement Information System (NIH PROMIS) Cancer Pain Interference measures (a composite score assessed at each palliative care clinic visit to identify patients experiencing difficult pain) [38,39], and (4) a primary informal (unpaid; family, defined broadly) caregiver who helped manage their care and symptoms at home. Both patients and caregivers were aged >18 years, English speaking, and did not have cognitive or visual deficits that would preclude the ability to participate in the study. Palliative care clinicians helped screen and verify the clinical eligibility of possible study participants.

### Data Collection Procedures

Before data collection, approval was granted by the University of Virginia Health Sciences Institutional Review Board. Both patients and caregivers provided informed consent. A study guide was created, informed by the literature and the research study aims (Multimedia Appendix 1). In addition to basic demographic questions, the study guide consisted of 3 parts.

#### Part 1

Part 1 consisted of open-ended questions regarding general challenges and concerns in managing cancer pain at home. Patients and caregivers were asked (1) Have you/the patient experienced cancer pain at home in the past week or so? If so, can you describe the experience from your perspective?; (2) What has been the most difficult part of managing pain at home?; and (3) What would help make managing the pain at home easier?

#### Part 2

Part 2 consisted of a list of variables that may influence cancer pain in the home setting that participants were asked to rank regarding impact. The list of variables was created based on their known relationship with cancer pain (such as the connection between sleep and pain) [40,41], current technological capabilities of the parent BESI system [42-44], and our hypotheses that certain environmental variables (eg, light and noise) that have received scant attention in the literature [26] can influence cancer pain. Overall, 14 variables were included in the final list and grouped into 4 categories: medication, wellness, interaction, and environmental. The primary objective of the variable list was to help inform the design of the BESI-C system (ie, which sensors to include) and validate our data collection plan.

Participants were asked to rate, on a Likert-type scale of 0 (no impact) to 5 (significant impact), the degree to which they thought each variable may influence the patient’s experience of cancer-related pain. For example, patients were asked how much they felt their mood or the temperature of the room impacted their pain on a scale from 0 to 5. Caregivers were presented with the same list of variables and asked to quantify how much each factor influenced the patient’s pain, *from their perspective* *as the caregiver.* Patients and caregivers were instructed that the study team was interested in their individual opinion and perspective and that it was fine if their answers differed from those of their partner. If a participant felt the *correct* answer was between 2 discrete values on the scale, they could indicate a half-way point, for example, 3.5. If participants remained unsure and did not feel they could quantify the impact of a particular variable, the item was skipped. (Note: participants were not asked about the direction of the variable [eg, does light make your pain worse?], but instead if they felt the variable impacted their pain, either positively or negatively [eg, how much of an impact, negative or positive, does light have on your pain?]). After the set list of variables was reviewed, participants were asked if there were any additional factors they felt influenced cancer pain at home that they were not asked about (eg, “Are there other factors we did not ask you about that you think influence the experience of cancer pain at home? What did we miss?”)

#### Part 3

**Figure 2:**
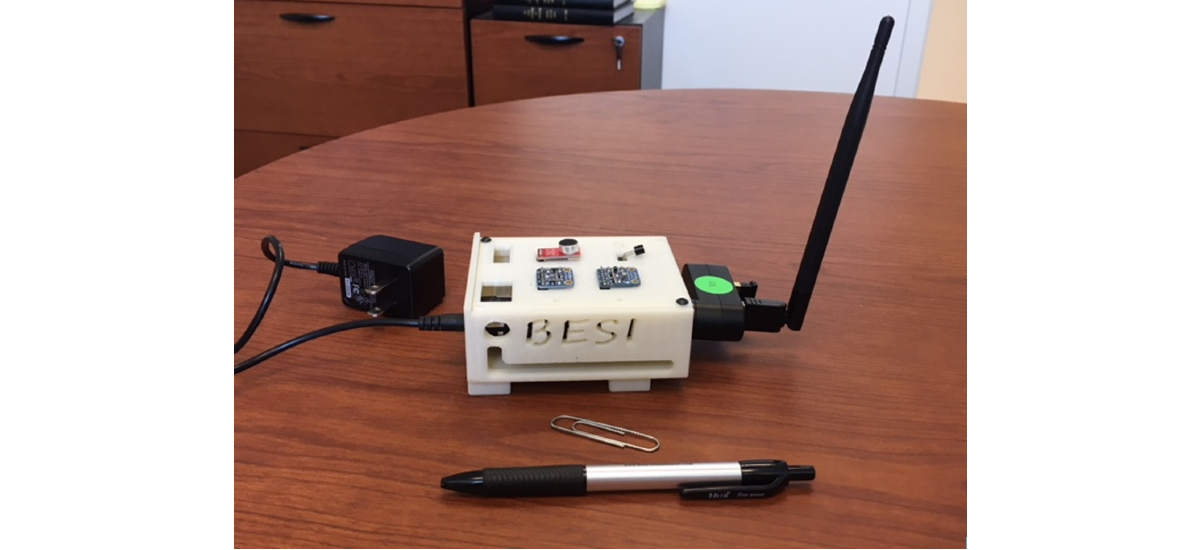
Behavioral and Environmental Sensing and Intervention for Cancer (BESI-C) initial environmental sensor.

**Figure 3:**
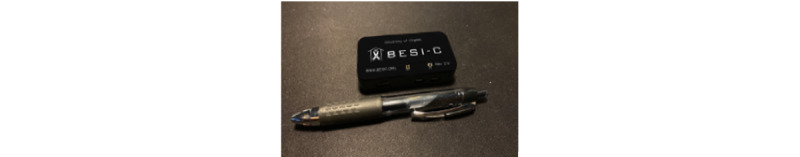
Behavioral and Environmental Sensing and Intervention for Cancer (BESI-C) updated environmental sensor based on user design input.

**Figure 4:**
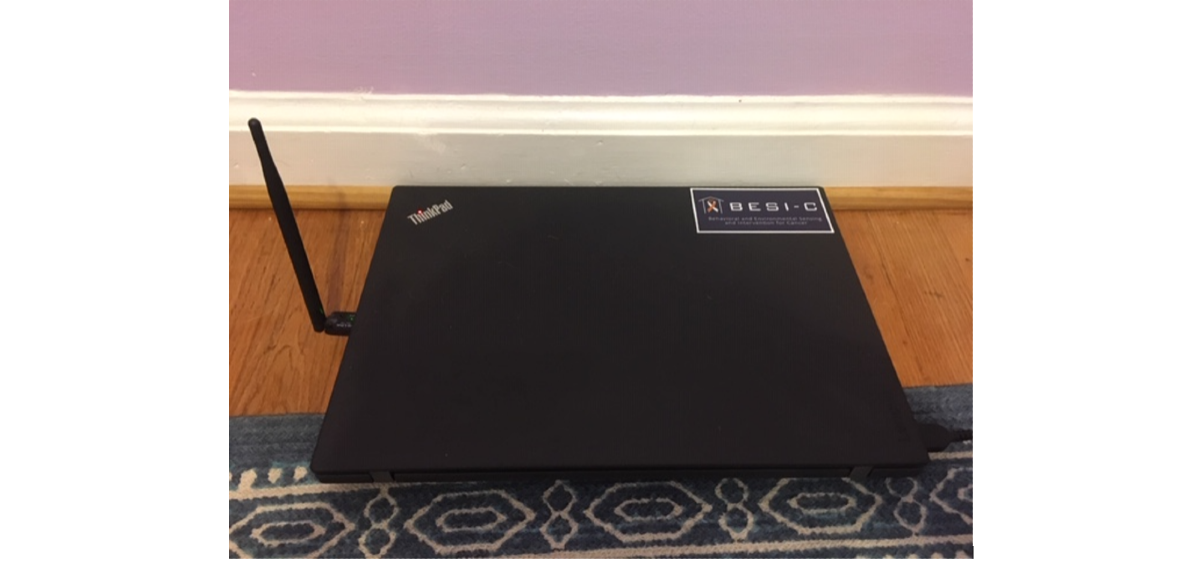
Behavioral and Environmental Sensing and Intervention for Cancer (BESI-C) base station laptop.

**Figure 5:**
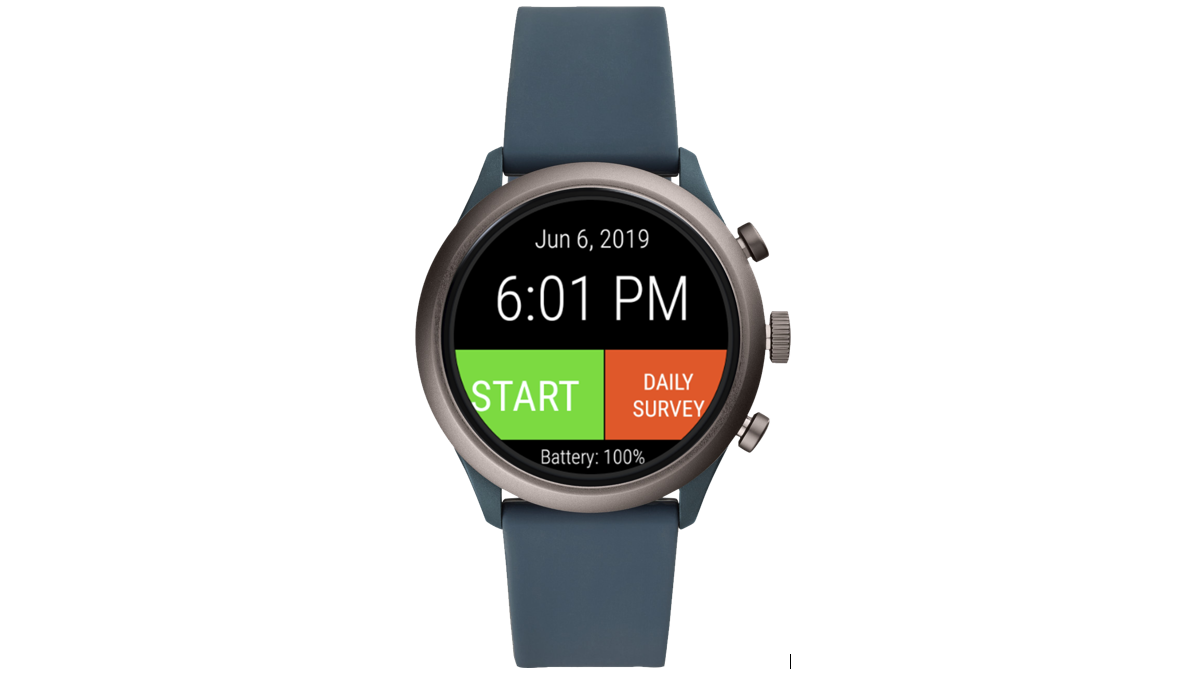
Behavioral and Environmental Sensing and Intervention for Cancer (BESI-C) smart watch with custom app.

Part 3 consisted of structured questions regarding the desired features of the BESI-C system. Participants were shown physical prototypes or pictures of the proposed components of the BESI-C system, including environmental room sensors, wearable sensors (smart watches), and a laptop base station used for remote system monitoring and local data processing and storage (Figures 2-5). Patients and caregivers were then asked about their general impressions, concerns, and suggestions regarding each system component. A key objective of this part of the interview was to ascertain how willing participants would be to interact with specific components of the system, for example, how often they would be willing to answer ecological momentary assessments (EMAs) [45] (brief survey questions) on a smart watch or if they had concerns about wearing a smart watch, in general. We were particularly interested in answering these specific design questions, as our goal was to create an unobtrusive smart health monitoring system that was acceptable, user friendly, and did not increase burden in an already highly stressed and vulnerable patient population.

Dyads were interviewed together or separately, generally in the outpatient palliative care clinic, based on their preference and logistic considerations. (Note: we recognize that this difference may have influenced participant responses, which is discussed in more detail below.) For convenience, caregivers who were unable to accompany the patient to the clinic were given the option to be interviewed over the phone (to complete part 3 over the phone, caregivers were provided with detailed verbal descriptions by the interviewer and/or pictures of the BESI-C components for visual reference). Interviews were audio-recorded with permission; detailed notes and responses were also recorded using pen and paper during all interviews by the research team member. Interviews lasted approximately 30 min, and dyads received a US $10 gift card as compensation for their time.

### Data Analysis Procedures

#### Qualitative Data

Interviews were transcribed verbatim and verified, and all identifiers were removed. All transcripts were read in entirety before analysis to understand the data set holistically. Open-ended responses (Parts 1 and 3 of the interview) were exported into Microsoft Word and organized by response to each corresponding interview question, by patient and by caregiver (eg, all patient responses to question 1 were grouped together, and all caregiver responses to question 1 were grouped together). A deductive, descriptive qualitative approach was then used to analyze interview responses across the entire data set from the perspective of patients and family caregivers. In keeping with our study aims, codes (or descriptive labels) were applied to portions of text that discussed the general experience of managing cancer pain at home and system design feedback to help identify patterns and key themes. For example, if a participant discussed fears related to pharmacological management of pain, this was coded with the straightforward label *medication concerns*. Themes were identified by considering both frequency of codes (how often a similar message was conveyed) and intensity of response (the strength of an articulated opinion, either negative or positive). Our goal with the analysis of open-ended responses was not to conduct qualitative analysis with a high level of abstraction, but instead, consistent with a descriptive approach, to stay close to our data and more concretely understand participant responses to each interview question [46].

#### Quantitative Data

Quantitative responses (part 2 of the interview) were entered into SPSS (v25.0), and basic descriptive statistics were run, including frequency counts and percentages for demographic data and means calculated (overall, patient, and caregiver) for individual and category (medication, wellness, interaction, and environmental) pain variable impact scores. Independent sample *t* tests were performed across all individual and category pain variables to assess for statistically significant differences (α set at .05) between patient and caregiver mean scores.

## Results

### Interviews

A total of 22 individuals were interviewed (22/22, 100%), including 10 patient-caregiver dyads and 2 individual patients, whose caregivers did not accompany them to the original clinic visit and were unable to be contacted after 3 attempts. A total of 5 dyads were interviewed together (dyads 6, 7, 9, 10, and 12), and 5 dyads were interviewed separately (dyads 1, 2, 3, 5, and 11). Of the 5 dyads interviewed separately, 2 caregivers were interviewed over the phone owing to logistic constraints. All other interviews were conducted face-to-face. Results are presented below by demographics and then by section of the interview guide (Parts 1, 2, and 3) for clarity.

### Demographic

Overall, almost half of the total sample (10/22, 46%) was aged between 50 and 59 years, with an equal number of females and males (11/22, 50%). The participants were primarily White (20/22, 91%) and non-Hispanic/Latino (21/22, 96%). The majority of patients (11/12, 92%) had a primary residence classified by the Centers for Medicare and Medicaid Services as rural [47]. Caregivers were predominantly female (7/10, 70%), lived full time with the patient (9/10, 90%), and were the significant other or spouse (5/10, 50%). The average patient-reported NIH PROMIS pain interference score was 7.16 (0, lowest interference to 10, highest interference), and half of the patients (6/12, 50%) self-reported their performance status as symptomatic, but ambulatory and able to complete their basic needs independently (Eastern Cooperative Oncology Group [48] score of 1). The most common malignancy was lung cancer (4/12, 33%), and 50% (6/12) of patients received their diagnosis <1 year ago. Table 1 presents the demographic data for the overall sample, patients, and caregivers. Table 2 presents cancer-related details for the patient sample.

**Table 1:** Demographic characteristics of the patient and caregiver sample.

Demographic variable		Total (N=22), n (%)		Patients (n=12), n (%)		Caregivers (n=10), n (%)	
**Age range (years)**							
	18-29		2 (9.1)		0 (0)		2 (20.0)
	30-39		2 (9.1)		1 (8.3)		1 (10.0)
	40-49		4 (18.2)		2 (16.7)		2 (20.0)
	50-59		10 (45.5)		7 (58.3)		3 (30.0)
	60-69		3 (13.6)		1 (8.3)		2 (20.0)
	>70		1 (4.5)		1 (8.3)		0 (0.0)
**Gender**							
	Female		11 (50)		4 (33.3)		7 (70.0)
	Male		11 (50)		8 (66.7)		3 (30.0)
**Race**							
	Black/African American		1 (4.5)		1 (8.3)		0 (0.0)
	White		20 (90.9)		11 (91.7)		9 (90.0)
	Missing (not asked)		1 (4.5)		0 (0)		1 (10.0)
**Ethnicity**							
	Latino/Hispanic		0 (0)		0 (0)		0 (0.0)
	Non-Latino/Hispanic		21 (95.5)		12 (100)		9 (90.0)
	Missing (not asked)		1 (4.5)		0 (0)		1 (10.0)
Rural^a^		N/A^b^		11 (91.7)		N/A	
**Highest education level**							
	Less than high school		5 (22.7)		3 (25)		2 (20.0)
	High school graduate		8 (36.4)		4 (33.3)		4 (40.0)
	Some college		2 (9.1)		1 (8.3)		1 (10.0)
	2-year degree		2 (9.1)		2 (16.7)		0 (0.0)
	4-year degree		4 (18.2)		2 (16.7)		2 (20.0)
	Professional/graduate degree		1 (4.5)		0 (0)		1 (10.0)
	Doctorate		0 (0)		0 (0)		0 (0.0)
**Current employment**							
	Full time		4 (18.2)		0 (0)		4 (40.0)
	Part time		1 (4.5)		1 (8.3)		0 (0.0)
	Retired		5 (22.7)		2 (16.7)		3 (30.0)
	Unemployed		12 (54.5)		9 (75)		3 (30.0)
**Caregiver lives with patient**							
	Yes, full time		N/A		N/A		9 (90.0)
	Yes, part time		N/A		N/A		1 (10.0)
**Caregiver relationship with patient**							
	Significant other/spouse		N/A		N/A		5 (50.0)
	Sibling		N/A		N/A		1 (10.0)
	Parent		N/A		N/A		1 (10.0)
	Child		N/A		N/A		2 (20.0)
	Other (daughter-in-law)		N/A		N/A		1 (4.5)

^a^Rural determined by the Centers for Medicare and Medicaid Services based on patient’s address of primary residence.

^b^Not applicable.

**Table 2:** Patient sample cancer characteristics (N=12).

Patient cancer variable		Total, n (%)
**Primary cancer diagnosis**		
	Breast	1 (8)
	Gastrointestinal (other)	1 (8)
	Gastrointestinal (pancreatic)	1 (8)
	Gynecological	1 (8)
	Head and neck	2 (17)
	Hematological^a^	1 (8)
	Lung	4 (33)
	Prostate	1 (8)
**Time since diagnosis (years)**		
	<1	6 (50)
	1-5	4 (33)
	5-10	1 (8)
	>10	1 (8)
**Patient self-reported ECOG^b^ score**		
	0, normal activity	0 (0)
	1, symptomatic and ambulatory	6 (50)
	2, ambulatory 50%, some help needed	2 (17)
	3, ambulatory <50%, nursing care needed	3 (25)
	4, no self-care, bedridden	0 (0)
	Not available	1 (8)
NIH PROMIS^c^ pain interference score^d^ 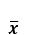 (n)		7.16 (12)

^a^Multiple myeloma.

^b^ECOG: Eastern Cooperative Oncology Group; standard patient performance scale.

^c^NIH PROMIS: National Institutes of Health Patient-Reported Outcomes Measurement Information System.

^d^Patient self-reported NIH PROMIS pain interference composite score, scored for clinical use on a scale of 0 (least) to 10 (most).

### Part 1: Understanding the Experience of Managing Cancer Pain at Home

Patients and caregivers uniformly described the experience of managing cancer pain at home as stressful and difficult. Key themes included (1) unpredictability and perceived inevitability of pain episodes; (2) impact of pain on daily life, especially the negative impact on sleep, activity, and social interactions; and (3) concerns regarding medications. All 3 themes overlapped as they did not occur in isolation. For example, the unpredictability of pain episodes could be worse at night when pain medications did not seem to be as effective, thus affecting sleep. When asked what could make managing pain at home easier, one caregiver simply stated, “when he heals, and this goes away,” (CG1). Others suggested ideas such as more rapid-acting interventions to relieve pain; reduced back-and-forth travel for medical appointments; a more holistic, nonpharmacological approach to managing pain; and better ways to track and record medication use. Textbox 1 summarizes and presents exemplar quotes related to part 1 of the interview.

**Experience of managing cancer pain at home from the perspective of patients (Pt) and caregivers (CG).**

**What is the experience of managing cancer pain at home? What is most difficult?**

**Theme 1: Unpredictability and perceived inevitability of pain**

- “That’s one thing about the cancer pain, is that you never know what you’re going to experience.” Pt 5
- “I do about all I can do. I don’t see it being any easier. It just stays, you know, it’s going to be what it’s going to be. It’s not going to get any better or any worse.” Pt 7
- “No, I don’t think you can manage the pain.” Pt 9
- “It hits me so bad sometimes it brings tears to my eyes…When I’m in really, really bad pain it gets me down.
- I get depressed and it’s like, ‘God, is this ever gonna quit?’” Pt 11
- “Well, I know it hurts. Some days it looks worse than others.” CG 2
- “The experience to me, he gives me a headache,” CG 1

**Theme 2: Impact of pain on daily life**

- “Well, you know, I'm miserable... I pretty much became a hermit since this happened. You know, I try to stay away from everybody, so I don't have to talk very much. I stay in the bedroom, you know, and watch TV most the time so I don't have to talk to people.” Pt 1
- “The intensity is worse in the evening at night...and also when I wake up in the morning. [The pain pills] are less effective at night...Sleep [is the most difficult part]. It’s most frustrating when it [pain] has kept me awake or wakes me up.” Pt 3
- “I’m an active person and with the pain I could barely get my shirt off.” Pt 4
- “Miserable. Miserable, I don’t do nothing. I can’t.” Pt 9
- “The bottom line is just be secluded when I am in pain...When I’m really, really in pain, if I’m alone it seems to soothe it…nothing there to irritate me to make it worse.” Pt 11
- “He was in real agony for a couple weeks, so bad he couldn't sleep.” CG3
- “I find it a little difficult...like she appears to be in pain, definitely lethargic and I think between the pain and feeling tired that definitely affects her mental health...so it’s just kind of all blurred together.” CG 5
- “It’s really pulled her down. You know, we went from being outside every day and doing things to, you know, pretty much watch watching her lay on the couch.” CG 9

**Theme 3: Concerns regarding medications**

- *Fear of running out of medication or becoming addicted*
  “I’m concerned that somebody will say you can’t have it anymore when I still need it. I know they’re addictive...but for me they’re necessary for the pain.” Pt 3
  I haven’t looked it [my pain medication] up on-line but I kind of worry about how dangerous it is for the rest of my body.” Pt 4
  “The only thing that changes my pain is my medication. Especially if I got it, I use it right, it makes a big difference. But when I run out, well, I got problems.” Pt 8
  “That’s my biggest fear is getting addicted.” Pt 9
  “I don’t really have enough medications, I guess...I take them just as they are prescribed to me...it’s frustrating after a while. Either I won’t be able to sleep, do I want to be in pain or do I want to conserve the medications and if I’m gonna have enough or God forbid I lose some or whatever...I ask for some [pain medication], ‘No.’ I ask again. ‘No.’ I don’t even ask them [health care providers] anymore. I’m tired of it. I feel like a little child asking for a piece of f*...ing candy. It’s frustrating as hell.” Pt 10
  “...he could be in a whole lot less pain, but that’s regulation...What makes it difficult is that he knows that if he took another half a tablet he would be in less pain but if he takes that half a tablet extra than he’s gonna have to be in more pain later [because there are not enough tablets].” CG 10
- *Coping with side effects*
  “They’ve had me on so many different medications and you can take 30 pills a day and still not get the relief you need…and that’s hard on your body. You’re dealing with all the different side effects...” Pt 5
  “...a lot of times I think I’d almost prefer to be in pain sometimes and live a little bit of life than sleep my life away.” Pt 10
  “Approaching things from a more holistic point [would help]...it seems that it’s just very much medication based and then side effects and then you treat those side effects with medications and then those side effects with other medications...” CG 5
  “I don’t think any of it is doing its job. I mean, it did at first, but I think that her body’s just gotten so used to it and it’s not doing what it was doing.” CG 9
- *Keeping up with, and keeping track of, medications*
  “Managing my pain, being on so many different medicines for pain, trying to make sure I take them all...it’s time consuming.” Pt 1
  “Most difficult? Taking my medicine. Sometimes I’ll take more than I should if I’m really in a lot of pain, and I know I’m not supposed to but it’s hard not to.” Pt 2
  “It [oxycodone] eases it a heck of a lot...I can sit and relax once it kicks in but once it wears off I’m going right back in the same state again. The pain comes back...Then I say when it gets to the point where I just take one pill to kill the pain, fine, but taking two, you know, it could be a problem.” Pt 11
  “I don’t always ask when I need it [pain medicine].” Pt 6
  “I can see when you need it [pain medicine] but I don’t just automatically give it to you...you’ve got to ask for it too…[it would be easier] if [my husband] would ask for [pain medicine] instead of me saying, ‘do you need your pain medicine?’” CG 6

**What would make managing pain at home easier?**

- “Having something that would provide instantaneous relief because sometimes it just seems like it takes a long time for anything to take effect.” Pt 3
- “Not having to keep going back and forth to the doctor so many times. It hurts her riding in the car.” CG 2
- “Some way to track when you’ve actually taken something because he’s writing it down, but when he was really dopey, he either didn’t remember to write it down or he couldn’t read what he wrote because he was so doped up. Something that would, I don’t know what kind of technology there would be, but something that scans the pill bottle or something and says ‘Ah, you’ve taken another one, so great, good for you’.” CG 3

The unpredictability of pain manifested in both the timing of the pain, which could occur abruptly and severely, and the lack of clarity regarding the origin of the pain. Patients often had multiple potential sources of pain, such as rheumatoid arthritis and cancer; this made it difficult for participants to sort out which pain was related to cancer and which was not, and then how to best and most appropriately intervene. The impact of pain on daily life was particularly noted in the areas of sleep, activity, and social engagement. Patients and caregivers reported a vicious cycle related to pain and sleep: pain intensity could flare up at night, causing insomnia, which resulted in worsening of pain and social withdrawal. Some participants, primarily patients, expressed a fatalistic attitude that cancer pain is inevitable and inherently unmanageable, regardless of what they tried to do to alleviate or mitigate the pain.

A strong theme in the interviews was related to challenges regarding pharmacological management of pain. Both patients and caregivers gave specific examples detailing the significant labor—both logistic and emotional—involved in managing cancer pain medications. Logistically, managing pain involved time and discomfort of multiple trips to the clinic for medical appointments; vigilance to coordinate, monitor, and remember complex and ever-changing pain medication regimens; *keeping ahead* of the pain by remembering to take pain medications before the previous dose wears off; and coping with, and balancing, side effects such as the perceived tradeoff between having pain better controlled but becoming too drowsy. Emotionally, participants discussed frustration and deep fears about running out of prescription opioid pain medications, being unable to obtain needed refills, or becoming *addicted*. These fears often resulted in the rationing of tablets, further exacerbating pain and distress. Medications were viewed by both patients and caregivers as an essential, but imperfect, tool that offered temporary relief from the pain, but that came with a (metaphorically) high price tag.

### Part 2: Variables that Influence Cancer Pain at Home

Table 3 compares the mean impact scores (0, no impact; 5, highest impact) for factors that may influence a patient’s pain at home by *category* (medication, wellness, interaction, and environmental). Table 4 presents the ranking of the *individual* variables from 1 (highest scored factor) to 14 (lowest scored factor) by comparing mean impact scores by the overall sample, patient, and caregiver. No statistically significant differences between patient and caregiver mean scores were detected across all variables.

Overall, and for patients and caregivers, *taking pain medication* was rated as the category with the highest impact on a patient’s pain (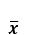
=4.79), followed by the categories *wellness* (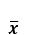
=3.60; sleep quality/quantity, physical activity, and mood and oral intake) and *interaction*, (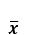
=2.69; busyness of home, social/interpersonal interactions, physical closeness/proximity to others, and emotional closeness/connection to others). The category related to *environmental* factors (temperature, humidity, noise, and light/brightness) was rated with the lowest overall impact (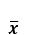
=2.51).

Regarding individual variables within each category, in the wellness category, the individual variables of sleep quality, sleep quantity, and physical activity were rated as having the most impact on a patient’s pain by the overall sample (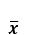
=4.28, 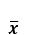
=3.98, and 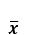
=3.90, respectively), by patients (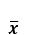
**=**3.91, 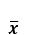 1=3.96, and 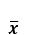
=3.91, respectively), and by caregivers (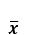
=4.72, 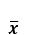
=4.00, and 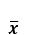
=3.89, respectively). In the interaction category, the variable busyness of home was rated with the highest impact score by the overall sample (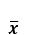
=3.21) and by caregivers (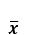
=3.28). Patients rated *social and interpersonal interactions* as having the highest impact on their pain (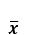
=3.50). In the environmental category, temperature was the highest rated variable by the overall sample (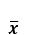
=3.30) and by patients (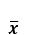
=3.63); caregivers rated humidity as the highest impact environmental variable (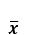
=3.22).

The rank order of individual variables (Table 4) revealed that pain medication, sleep quality/quantity, physical activity, and mood occupied the top 5 spots for the overall sample and for both patients and caregivers. Other variables related to environmental and contextual factors were ranked more diversely. Interestingly, emotional closeness/connection to others was the lowest ranked variable by patients, whereas *noise* was the lowest ranked for caregivers (but #9 for patients).

When asked if there were other variables that influenced cancer pain not included on our list that we should measure with a home monitoring system, only 2 participants identified additional variables. One caregiver (CG 1) stated that the amount the patient talks influenced his pain (patient had a diagnosis of head and neck cancer) and felt this was an important variable to assess. One patient (Pt 4) added “good support group of people to help” as a broader interpretation of our questions regarding the impact of emotional connection and social interaction. A total of 3 patients responded to this question by reiterating the importance of pain medication as the most important variable that influenced their cancer pain.

**Table 3:** Comparison of mean impact scores of factors that influence a patient’s cancer pain at home, by category and individual variable, rated from 0 (no impact) to 5 (highest impact).

Category and individual variables^a^		Overall (N=22), n (%)	Patients (n=12), n (%)	Caregivers (n=10), n (%)
		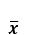 (n^b^)	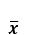 (n)	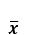 (n)
**Medication, category mean**		4.79	4.79	4.78
	Taking pain medication	4.79 (21)	4.79 (12)	4.78 (9)
**Wellness, category mean**		3.60	3.45	3.80
	Sleep quality (how well)	4.28 (20)	3.91 (11)	4.72 (9)
	Sleep quantity (how much)	3.98 (21)	3.96 (12)	4.00 (9)
	Physical activity	3.90 (20)	3.91 (11)	3.89 (9)
	Mood	3.45 (20)	3.55 (11)	3.33 (9)
	Oral intake (eating/drinking)	2.43 (20)	1.91 (11)	3.06 (9)
**Interaction, category mean**		2.69	2.82	2.52
	Busyness of home	3.21 (19)	3.15 (10)	3.28 (9)
	Social/interpersonal interactions	2.97 (16)	3.50 (9)	2.29 (7)
	Physical closeness/proximity to others	2.38 (21)	2.73 (11)	2.00 (10)
	Emotional closeness/connection to others	2.20 (20)	1.90 (10)	2.50 (10)
**Environmental, category mean**		2.51	2.48	2.50
	Temperature	3.30 (22)	3.63 (12)	2.90 (10)
	Humidity	2.61 (18)	2.00 (9)	3.22 (9)
	Noise	2.07 (21)	2.21 (12)	1.89 (9)
	Light/brightness	2.05 (20)	2.08 (12)	2.00 (8)

^a^Instructions provided to participants during the interview: Please think back over the past few weeks or months. Patient: for each item, on a scale of 0-5 (0=not at all, 5=a great deal), how much do you think it makes your pain better or worse? Caregiver: for each item, on a scale of 0-5 (0=not at all, 5=a great deal), how much do you think it makes the patient’s pain better or worse?

^b^Where “n” is not equal to the total sample, participant either was unsure/could not answer or the item was not asked (social/interpersonal interaction factor question was added after dyad 3).

**Table 4:** Rank order of individual variable impact means (0=no impact; 5=highest impact) on patient’s pain.

Rank	Overall		Patient		Caregiver		
	Variable	Mean	Variable	Mean		Variable	Mean
1	Pain medication	4.79	Pain medication	4.79		Pain medication	4.78
2	Sleep quality	4.28	Sleep quantity	3.96		Sleep quality	4.72
3	Sleep quantity	3.98	Sleep quality (tie); physical activity (tie)	3.91		Sleep quantity	4.00
4	Physical activity	3.90	Temperature	3.63		Physical activity	3.89
5	Mood	3.45	Mood	3.55		Mood	3.33
6	Temperature	3.30	Social/interpersonal interactions	3.50		Busyness of home	3.28
7	Busyness of home	3.21	Busyness of home	3.15		Humidity	3.22
8	Social/interpersonal interactions	2.97	Physical closeness/proximity to others	2.73		Oral intake	3.06
9	Humidity	2.61	Noise	2.21		Temperature	2.90
10	Oral intake	2.43	Light/brightness	2.08		Emotional closeness/connection to others	2.50
11	Physical closeness/proximity to others	2.38	Humidity	2.00		Social/interpersonal interactions	2.29
12	Emotional closeness/connection to others	2.20	Oral intake	1.91		Physical closeness/proximity to others (tie)*;* light/brightness (tie)	2.00
13	Noise	2.07	Emotional closeness/connection to others	1.90		Noise	1.89
14	Light/brightness	2.05	N/A^a^	N/A		N/A	N/A

^a^N/A: not applicable.

### Part 3: Feedback Regarding the BESI-C System Components

The results presented in Textbox 2 focus on the 2 primary components of the BESI-C system: environmental and wearable (smart watch) sensors as well as general system impressions, suggestions, and concerns. (Participants expressed minimal or no concerns about the laptop base station, which we are currently removing from the system architecture and replacing with a cloud-based service for a simpler and less-intrusive system deployment and to facilitate more efficient data management.) Overall, patients and family caregivers expressed interest and receptivity to the concept of BESI-C, validated the importance of monitoring cancer pain at home, were eager for innovative ways in which to do so, and provided constructive feedback regarding the system components. However, there was the acknowledgment that providing feedback would involve actual use and pilot testing of the system. There was also the acknowledgment that a system such as BESI-C could be particularly helpful in assisting caregivers to *tune in* to variables that may influence a patient’s pain, but that may not be readily obvious. Participants expressed a strong preference for technology that is unobtrusive, simple, convenient, durable, and aesthetically pleasing and that involves minimal interference with daily activities, such as sleep. For both environmental and wearable sensors, participants expressed concerns regarding privacy and a desire for multifunctionality (eg, could environmental sensors measure variables beyond those focused on cancer pain, such as general air quality in the home, or other symptom management issues, such as sleep apnea, and could wearables measure additional factors such as blood pressure or blood glucose levels).

**Feedback from patients and caregivers regarding the Behavioral and Environmental Sensing and Intervention for Cancer system components.**

**General impressions and interest**

- “I think it’s exciting that somebody’s coming up with this. I really do...the information that you can get from the sensors and the watch.” Pt 5
- “I think what you showed me is a good idea.” Pt 8
- “I do too [think it would be cool] because anything to try to help stabilize the pain.” Pt 9
- “Anything you all can come up with to help, I’d approve of anything. Yes, I would, because cancer is bad. It’s very painful... It’s just terrible.” CG 2
- “No big deal, we like stuff like that...it’s useful.” Pt 7
- “Those are things that even if it’s a question that you just ask, those are things that we don’t pay attention to every day. You know, the stress level in a room, issues like that. We’re not really tuned in to that.” CG 9

**Environmental Sesnors**

- *Importance of household buy in*
  - “Wouldn’t bother me, but may bother my Dad...if it transmits information somewhere else and it monitors stuff in his house he probably wouldn’t like it.” Pt 1
  - "That would be good to me...It might not bother me, but it’s going to bother her [caregiver].” Pt 8
  - “With me, if this is only gonna be in my room, fine. It wouldn’t bother me one bit. But as far as the one in the kitchen, the living room, her [daughter in law’s] bedroom, I don’t think she’s gonna agree with that...You just got to figure out if we’ve got enough outlets to put these things in that won’t interfere with her cooking, appliances, stuff like that... what I’m saying is run it by her and see what she thinks.” Pt 11
- *Desire for multifunctionality*
  - “It reminds me of a little robot...that’s wonderful, so I’m glad if it will work. Could you put a smoke detector in there?” Pt 4
  - “Well, with his condition, I mean it is a good idea for something like that, not just the cancer, with his sleep apnea and all that stuff and like his asthma and stuff. I mean, that would be a good idea [to monitor too].” CG 1
  - “I think that’s interesting. I would like to know exactly, you know, what I’m breathing, and you know, the air and stuff in the house.” Pt 2
  - “Is there a way to monitor diet or when someone’s eating or not eating? I just know with my mom sometimes when she’s feeling a lot of pain, she could go an entire day without eating.” CG 5
- *Privacy considerations and data sharing*
  - “Cool. I’d want to know–okay, so it measures all of that–then what does it do with it? Does it spit it out at the doctor’s office? Can you get it through an app? Can you look at what it’s doing?” CG 3
  - “As long as it ain’t watching us.” CG 9
  - “My major concern would be the privacy.” Pt 9
  - “I would be quite concerned if it’s recording what I’m saying.” Pt 10

**Smart watch**

- *Desire for simplicity and comfort*
  - “Should be super simple like the old people’s cellphone, the Jitterbug. I get up in the middle of the night and I don’t have my glasses on...so it’s got to be really self-explanatory. Like you look at it, and you go, ’Red is bad, green is good’.” CG 3
  - “I don’t like jewelry on me and stuff on my wrist...working on cars and stuff, a watch gets in the way.” Pt 1
  - “I’d be concerned about how comfortable it is, how easy it would be to put on, about finding it if I took it off ‘cause I tend to lose things like watches.” Pt 3
  - “I see it possibly interfering just with work maybe. Just because of the work that I do [manual labor]. But other than that, I mean I think on her it can be beneficial.” CG 5
  - “I wouldn’t want it to take up my life, but I would be willing to try it.” Pt 4
- *Privacy considerations and interfacing with the technology*
  - “People our age, it’s stereotypical, but it’s way too small. I cannot imagine trying to answer a question on that.” CG3
  - “I mean she [the patient] could wear it, but I don’t know if I could, to be honest with you. When it comes to this high tech stuff I don’t know nothing about it. I wouldn’t mind wearing it, if I could learn how to work it. You know, I would love to do it.” CG 2
  - “As long as it’s not picking up on my personal stuff, I wouldn’t have a problem with it...I just don’t want my personal life put out there.” Pt 9
- *General impressions of smart watch *
  - “I think it’s a good idea myself. I think that would make a big different in monitoring some things.” CG 6
  - [Could be tricky to remember to mark pain events]: “Would it give like an alert or something or some noise? I think once I get used to it I think it would be great [and I would remember]” Pt 2
  - [Willing to answer questions multiple times a day]: “whenever I needed to.” CG 11
  - “It would be an annoyance to you because you’d have to answer it all the time...It does look nice, though. It looks nice and sleek.” CG 12
  - “Convenient...doesn’t take any space up.” Pt 11
  - “I think it would be beneficial, but it would be a pain.” Pt 12

**Smart watch versus tablet to answer ecological momentary assessments (EMAs)**

- “The watch because it’s with me all the time, even when I’m not at home.” Pt 2
- "Prefer iPad...it just looks easier to use. I assume it’s bigger...I would find the watch the most inconvenient, because I’d always have to have it on or keep track of it or whatever. Something I could just put in one place and forget about would be better for me.” Pt 3
- “iPad may not be as accurate because you’re not going to remember everything after the fact.” Pt 4
- “I’d rather do the watch...it would be easier for me...this is attached.” Pt 5
- “I think I’d like the tablet more but that’s me.” Pt 6
- “It [the watch] would be a lot easier for me...I’m no electronic expert, you know what I’m saying? I don’t deal with computers...[willing to interact with watch multiple times a day] anytime they [EMAs] popped up, as long as I’m not asleep.” Pt 11
- “[I’d prefer the watch] because I can’t stand an iPad. I had one at work and I just could not.” Pt 12
- “I think having a watch, having it all together in one unit would just be more streamlined so you don’t have to keep up with multiple devices.” CG 5
- “Probably for a lot of people it would be easier on the tablet, but my only thought to that is...would somebody actually go for the tablet and answer it? You know, if it’s on the watch you would do it automatically cause it’s right there.” CG 6
- “Either one is fine with me...in the summertime I really don’t want a tan line...so I would want to take a watch off if I had to wear it.” CG 11

**System suggestions/concerns**

“You could set intervals at different times to remind you to take different types of medicine at different times; [that] would be about the best thing I know.” Pt 1
“For older people, as simple as possible. When you get to be a lot older you really don’t want to have to fuss with a lot of things that aren’t central to your condition...if you’re in pain this kind of stuff’s going to go out the window, so I should say as simple as possible, absolutely as simple as possible.” CG 3
“We just have really horrible internet...our internet is just off of a hotspot from my cell service. That’s the only internet we have.” CG 5
“The only concern I would have is, is it going to be like making noise and stuff like that?” CG 11
“Those are things that even if it’s a question that you just ask, those are things that we don’t pay attention to every day. You know, the stress level in a room, issues like that. We’re not really tuned in to that.” CG 9
“I’ve got to see what all we gonna have set up in there. I’ve gotta see how comfortable I am with this by seeing how it all works, you know, and then I can give you my opinion, based on everything.” Pt 11

**Dyadic effect of technology**

“I think most people it wouldn’t bother. For me it’s different. I don’t like jewelry.” [Pt 6–then after hearing wife’s receptiveness to the watch said:] “I think it would [make a difference]. I would make myself wear it if I had to.” Pt 6
“Yeah, she’s [caregiver] taking it kind of hard...” Pt 8, [when explained how the watch would help monitor CG too.]
“As long as she [patient] doesn’t mind, I wouldn’t mind.” CG 9
[Patient acknowledging importance of monitoring caregiver experience]: “I think he [caregiver] kind of puts on a show of handling it better than he does.” Pt 5

Specific to the environmental sensors, patients discussed the importance of having everyone in the home consent to sensor placement. Primary privacy concerns related to possible audio and video data collection as well as data sharing (eg, where are the data going and who has access to them). Participants also discussed practical issues related to internet connectivity and power outages (particularly in rural areas); the number of electrical outlets in the home needed to plug in environmental sensors; how much space the sensors would occupy; and placing sensors so that they are discrete, out of the way of small children, and any cords are safely secured to prevent trip hazards or falls.

Regarding wearable sensors, participants were intrigued by the smart watch platform but wondered if (1) it would be difficult or complicated to answer questions on the watch owing to the relatively small touch screen, (2) they would have trouble using the technology, and (3) EMAs would become annoying. Some participants expressed concerns about having multiple watches and having to charge them and the potential for them being misplaced. Similar to environmental sensors, concerns were expressed regarding privacy and data sharing (eg, what exactly is being collected and where are the data going and when), and some participants whose jobs required manual labor were concerned that the watch may interfere with their work or described themselves as individuals who just did not like wearing watches or *jewelry*. Participants emphasized the importance of a simple, clear user interface, and all but 1 participant reported a willingness to answer EMAs on a wearable device. A total of 85% (17/20; 2 participants not asked) of the participants reported that they would prefer to mark and characterize pain events on a smart watch compared with a tablet, as the watch would be *attached* to them and they would be less likely to forget important details (Table 5). Some participants felt that answering EMAs on a wearable device could be annoying and they may forget to do it, but others were willing to answer as many EMAs on the smart watch as needed.

One interesting finding involved divergent perceptions between patients and caregivers regarding aspects of the BESI-C system. When patients and caregivers were interviewed together, caregivers often helped encourage an initially skeptical or reluctant patient to try the technology or reassured them about practical aspects, such as where environmental sensors could be placed in the home or that they actually had enough electrical outlets. When interviewed separately, patients commonly expressed concerns about the technology they *thought* caregivers would have, but which the caregiver did not actually express when interviewed independently. In addition, conversations related to the technology often revealed additional dyadic dynamics, beyond what was expressed regarding the impact of general symptoms. For example, when patients were asked about specific aspects of the technology, it often prompted comments acknowledging the difficulty of their illness on their caregiver and their concern for the impact it has had on them. Patients expressed that the BESI-C system would be helpful in providing an objective picture of how their caregiver was actually coping and what support their caregiver needed.

**Table 5:** Participant preferences and concerns regarding a wearable device to answer ecological momentary assessments.

Participant	Willing to answer EMAs^a^ on a wearable device, in general	Preference for tablet/smartphone versus smart watch for EMAs	Specific comments/concerns
Pt^b^ 1	May be	No preference	Tablet would have to have durable case; watch needs to be unobtrusive
CG^c^ 1	Yes	No preference	—^d^
Pt 2	Yes	Watch	Watch would be “awesome”
CG 2	Yes	No preference	Concern about ability to manage technology; would need to be easy
Pt 3	No	Tablet	Concerns about watch: comfort, loss, and potential burden
CG 3	Yes	Tablet	Worried about display size/visibility of watch and ease of button use on watch; worried about loss of watch
Pt 4	Yes	Watch	Concern about watch bulkiness versus size display
Pt 5	Yes	Watch	—
CG 5	Yes	Watch	Concern about wearing at work
Pt 6	Yes	Tablet	—
CG 6	Yes	Watch	—
Pt 7	Yes	Not asked	“Watch is high-tech, I like it”
CG 7	Yes	Not asked	—
Pt 8	Yes	Watch	Thinks CG would prefer tablet
Pt 9	Yes	Watch	Concerned about privacy; concerned about ability to be “outdoorsy”
CG 9	Yes	Watch	—
Pt 10	Yes	Watch	“I don’t want to answer to anybody”
CG 10	Yes	Watch	—
Pt 11	Yes	Watch	Concern about sleep interruption
CG 11	Yes	No preference	—
Pt 12	Yes	Watch	Privacy concerns
CG 12	Yes	Watch	Privacy concerns

^a^EMA: ecological momentary assessment.

^b^Pt: Patient.

^c^CG: Caregiver.

^d^No additional comments provided.

## Discussion

### Principal Findings

This research contributes to a more complete understanding of the experience of cancer pain in the home context and adds an important dimension of considering the caregiver’s perspective. It also fills an important gap in the evidence-based design of smart health monitoring and intervention systems to provide symptom support for patients and caregivers coping with advanced cancer, a population with often significant, and unmet, symptom management needs [35,49,50]. Our overall sample, although equally split between males and females, had a disproportionate number of female caregivers and, although not racially or ethnically diverse, represents a geographically underserved sample, as the majority of patients were from rural areas of Central Virginia (consistent with the general demographics of the cancer center recruitment study site). We recruited a final sample size of 22 individuals, which is consistent with the pilot, qualitative, and early stage smart health design work [33,51-60] and the aims of our study to explore proof of concept of the BESI-C system with end users and better understand the experience of cancer pain at home.

### Experience of Cancer Pain at Home: How This Can Inform System Design

Our key qualitative themes that highlight the unpredictability and perceived inevitability of pain, the negative impact of pain on foundational aspects of daily life (especially sleep, activity, and social engagement), and the challenges of managing and monitoring complex medication regimens are not surprising and validate a large body of knowledge regarding the difficulty of managing advanced cancer pain at home [4,9,10,15,23,24,61,62]. What is particularly noteworthy regarding concerns about medication management is the emphasis participants placed, especially patients, on fears and concerns regarding access to opioid pain medication, a mainstay therapy in the management of serious cancer pain. It is especially important to consider this finding in the context of the *opioid epidemic* [63], where increased scrutiny and stigma attached to opioid therapy, even for legitimate purposes, have created unintended and increased obstacles and barriers to pain relief [64,65]. This finding underscores the significant role of pharmacological strategies in the management of cancer pain at home and the importance of designing home-based monitoring systems equipped with capabilities to support patients and caregivers in tracking, monitoring, and using mediations safely and effectively, especially prescription opioids. Consistent with other literature [9,66-70], our interviews revealed that keeping track of changing and complex medication regimens is time consuming and stressful; home-based monitoring systems that can assist with this aspect of care are essential to optimally support patients and caregivers and should be thoughtfully designed to not contribute or exacerbate the stigma and fears associated with opioid therapy needed to treat legitimate cancer pain.

Another important finding from our interviews is the need for systems that capture the impact of unpredictable cancer pain that can escalate severely and without warning. This type of pain is commonly referred to as *breakthrough* pain and is notoriously difficult to manage [19,71,72]. Although patients and caregivers did not specifically use the term *breakthrough pain* during interviews, they described significant distress associated with abrupt pain of intense severity. Finding ways to effectively capture and characterize this type of rapid-onset pain requires systems that are simple, portable, and extremely quick and easy to use, and is a primary rationale for our interest in using wearable sensors to collect these data (vs smartphones or tablets). Breakthrough pain can be an *out of control experience* for patients and caregivers, resulting in feelings of hopelessness [19,71]. We suggest that a monitoring system designed to assist patients and caregivers in tracking, recording, characterizing, and, ultimately, treating breakthrough cancer pain episodes is vital to help restore a sense of control and empowerment over their situation.

In addition, although pharmacological management of cancer pain is critical to assess, participants also expressed a desire for a more holistic approach to managing cancer pain, especially given concerns regarding the multiple side effects of medications. We accounted for this finding by adding EMAs to our smart watch app that specifically asked about nonpharmacological approaches patients and caregivers use to manage cancer pain.

### Variables That Influence Cancer Pain: How This Can Inform System Design

A better understanding of the impact of variables that influence cancer pain in the home setting can facilitate the design of tailored systems equipped to measure and assess the most salient variables. Although we did not detect statistically significant differences between patient and caregiver mean impact scores (most likely due to our small sample size and the possible influence of participants being interviewed together), our results make an important contribution and extend existing work [30,73] as they (1) focus on perceived influences on cancer pain from *both* the patient and caregiver perspective; (2) consider a holistic set of environmental *and* contextual variables; (3) suggest important data collection features to include in remote symptom monitoring systems; and (4) provide initial insights into the impact of critical variables that may influence cancer pain, which can be built upon for future inquiry. The lack of a significant difference may suggest that patients and caregivers are largely *in sync* about what impacts pain, which could be helpful and productive. For example, both patients and caregivers ranked variables related to taking pain medication, sleep, activity, and mood among the top 5, and they both ranked *taking pain medication* as the most impactful variable influencing cancer pain. These findings corroborate our qualitative findings and again underscore the importance of designing monitoring systems that account for ways to support patients and caregivers in tracking and managing pain medications.

It is not surprising that sleep quality and quantity were rated, among all the wellness variables, as having the highest impact on pain as the relationship between sleep and pain has been well established [74]. Likewise, our results also confirm the known connection between mood and pain [75]. Patients and caregivers also rated physical activity as having a significant impact on patients’ pain, justifying the importance of including activity-monitoring features, such as pedometers and accelerometers. It is noteworthy that *sleep quality* is rated as the most impactful variable by caregivers, second only after taking pain medication. This underscores the need to design monitoring systems and interventions that do not further worsen or interrupt sleep, such as with low-battery reminders or bothersome sensor lights. Another particularly interesting finding within the category of wellness variables is the fact that caregivers rated the impact of oral intake (#8) higher than patients, who rated it as the second lowest rated variable (#12). For health care providers, this likely resonates as family caregivers often have strong opinions and beliefs/concerns about the nutritional intake of their loved ones, specifically how the lack of adequate oral intake may worsen distress and pain [76].

Interpreting the impact of interaction and environmental variables is more complex as there is less congruence between patient and caregiver scores. Regarding interaction variables, it is interesting that patients rated *emotional closeness to others/connection to others* as having the least impact on their pain, after noise, light, humidity, and oral intake. One interpretation of this finding is that patients do not value emotional connection or closeness or see little association in its role to their experience of pain, which seems unlikely. An alternative explanation could be that because we interviewed dyads (and most commonly spouses), emotional closeness/connection was assumed by the participants and therefore not considered to be a significant variable. If we had interviewed single patients with cancer, who may experience more notable fluctuations in available emotional support, this variable may have been rated differently. Another hypothesis, consistent with the principles of Maslow’s hierarchy of needs, is that patients experiencing significant pain will naturally focus first on the physical aspects of their well-being (such as sleep), with less priority given to higher level needs, such as emotional connection. However, patients and caregivers did rank *social/interpersonal interactions* and *busyness of home* higher on the list, suggesting that engagement within the home is important and can influence pain. These findings suggest that capturing the degree of social engagement within the home is important and should be incorporated into home monitoring systems with features that can track location and person-to-person interactions.

As a category, environmental variables were ranked as having the least influence on pain. However, as individual variables, patients ranked *temperature* as having the fourth highest impact, but all other environmental variables (noise, light, and humidity) were ranked in the bottom 5. For caregivers, all environmental variables were ranked in the lower half. One possibility is that environmental variables have little influence on a patient’s pain. Instead, we argue that environmental variables *are* likely to be important factors (just ask anyone whose pain increases during rainy, humid weather), but that patients and caregivers may simply not be fully aware of the role these variables play as they are rarely (if ever) systematically monitored, tracked, or reported, making their impact less obvious and more difficult to quantify. As monitoring environmental variables can be performed passively, requiring minimal participant burden, we suggest it is important to collect these data so that we can more clearly understand the potential relationships between environmental factors and pain episodes.

Finally, it is reassuring that participants did not readily identify additional variables to measure with the BESI-C system. In other words, patients and caregivers validated our list of proposed variables and felt that we included a comprehensive list.

### Feedback About the BESI-C Prototype Components: How This Can Inform System Design

Showing prototypes of the BESI-C system and discussing them with participants proved to be very effective and helpful in informing system design and refining the BESI-C system. For example, based on feedback from participants, we elected to use a smart watch to collect EMA data (vs a smartphone or table) and iterated our environmental sensors to make them smaller, sleeker, and more discrete. Another benefit of discussing the technology with participants is that it confirmed important dyad dynamics that reinforced our initial hypotheses about system design. For example, the ultimate goal of BESI-C is to improve communication between patients and caregivers, particularly around pain management. This was reinforced by comments from dyad 6, where the patient reported that he did not always ask for pain medication, even when he needed it, and the caregiver expressed that she was not always sure when the patient needed pain medication. Data from BESI-C could improve these types of interactions by providing helpful data in real time to patients and caregivers.

We paid particular attention to data privacy based on participant feedback. The system includes no cameras, and the microphone outputs are locally processed to extract relevant audio features (eg, loudness level and noise fluctuation) so that no interpretable audio data are stored or transmitted. Participants also had questions about data sharing and expressed interest in seeing their own data and having it be available to health care providers. Our initial pilot work does not fully explore this important question (although patients and caregivers will see selected extracted features of their data), but future work will examine how to optimally generate data visualizations and how to best share these data with relevant stakeholders.

Patients and caregivers expressed less concerns regarding passive monitoring and seemed to prefer elements of the system that could be left alone and just *do their thing*. This is important when considering the system design for this patient population and suggests that passive data collection, with environmental sensors or physiological monitoring with wearables, may be more acceptable and feasible and that active data collection with EMAs should be extremely judicious to reduce user burden. Simplicity and ease of use were critically (and not surprisingly) important to participants, and we designed our smart watch user interface to be extremely easy to use, intuitive, and to work well on a watch touchscreen [36]. Through all portions of the interviews, both patients and caregivers reinforced the importance of medication tracking and monitoring. On the basis of this feedback, the BESI-C EMAs include simple questions about medication use as well as reasons pain medication may not be taken even if patients are in pain (eg, concerns about running out of tablets).

### Limitations

The primary limitation of this study is the sample size, which reduced generalizability and precluded our ability to detect statistical significance in our analysis of variable impact scores. However, our sample size is consistent with the scope of a pilot study related to early stage smart health design [33,53,54] and the aims of qualitative research [51,52,56] and provides an important rural perspective. Our initial intent was to interview all dyads together, but this proved difficult/impossible due to logistical constraints. To avoid increasing the participant burden, a critical consideration for this patient population, some dyads were interviewed together and some separately. Although participants were instructed that we were interested in their own individual opinion and perspective, it is likely that for dyads interviewed together, hearing their partner’s responses may have influenced their answers. Finally, we do not know the direction of the impact of variables as we did not ask participants whether certain factors made pain better or worse, only whether they felt the variable had an important impact, either negative or positive. Therefore, for example, we cannot say that a high score of *physical activity* means physical activity improves pain or makes it worse—only that the variable of physical activity is perceived to have a significant influence on the patient’s pain.

### Future Directions

This study provides important foundational data that can inform future research, particularly related to understanding variable influence on the pain experience and how this can inform remote monitoring system design. Conducting a similar study in part 2 with a larger sample size of dyads would be helpful to detect statistically significant differences between how caregivers and patients rank variables that may influence pain. Relatedly, it would be interesting to explore whether, and how, concordance between variable ratings among patients and caregivers changes relevant clinical outcomes. For example, are dyads with higher variable congruence (eg, more agreement regarding the impact of certain variables that may influence pain) more likely to experience lower levels of pain and overall distress? This could be evaluated by asking patients and caregivers to independently score a list of variables, deploy a monitoring system such as the BESI-C, and then compare the reported pain and distress levels with predeployment variable congruence levels. Such results would help further inform the design of smart health monitoring systems and personalized interventions. In addition, more recent concerns regarding home-based care in the context of COVID-19 are prompting adjustments in the BESI-C system design to facilitate contactless deployments.

### Conclusions

Strategies to monitor and treat cancer pain outside the acute care setting are critical as most cancer symptom management occurs at home, often causing significant stress for both patients and family caregivers. Home-based smart health monitoring systems designed to support cancer pain management should account for the experience of both the patient and the caregiver; prioritize passive monitoring of physiological and environmental variables to reduce burden; and include functionality that can monitor and track medication intake and efficacy, wellness variables (such as sleep quality/quantity, physical activity, mood, and oral intake), and levels of social interaction and engagement. In addition, systems must consider concerns regarding privacy and data sharing and incorporate feasible strategies to capture and characterize rapid-onset symptoms.

## Appendix

Multimedia Appendix 1 Study guide.

## Abbreviations

BESI-C: Behavioral and Environmental Sensing and Intervention for Cancer
EMA: ecological momentary assessment
NIH PROMIS: National Institutes of Health Patient-Reported Outcomes Measurement Information System

## References

[1] van den Beuken-van Everdingen MH, de Rijke JM, Kessels AG, Schouten HC, van Kleef M, Patijn J (2007). Prevalence of pain in patients with cancer: a systematic review of the past 40 years. Ann Oncol.

[2] Goodwin PJ, Bruera E, Stockler M (2014). Pain in patients with cancer. J Clin Oncol.

[3] Deandrea S, Montanari M, Moja L, Apolone G (2008). Prevalence of undertreatment in cancer pain. A review of published literature. Ann Oncol.

[4] Smyth JA, Dempster M, Warwick I, Wilkinson P, McCorry NK (2018). A systematic review of the patient- and carer-related factors affecting the experience of pain for advanced cancer patients cared for at home. J Pain Symptom Manage.

[5] Teunissen SC, Wesker W, Kruitwagen C, de Haes HC, Voest EE, de Graeff A (2007). Symptom prevalence in patients with incurable cancer: a systematic review. J Pain Symptom Manage.

[6] Kutner JS, Kassner CT, Nowels DE (2001). Symptom burden at the end of life: hospice providers' perceptions. J Pain Symptom Manage.

[7] Phongtankuel V, Teresi JA, Eimicke JP, Kong JX, Adelman RD, Prigerson HG, Czaja SJ, Shalev A, Dignam R, Baughn R, Reid MC (2020). Identifying the prevalence and correlates of caregiver-reported symptoms in home hospice patients at the end of life. J Palliat Med.

[8] de la Cruz M, Noguera A, San Miguel-Arregui MT, Williams J, Chisholm G, Bruera E (2015). Delirium, agitation, and symptom distress within the final seven days of life among cancer patients receiving hospice care. Palliat Support Care.

[9] Mehta A, Cohen SR, Ezer H, Carnevale FA, Ducharme F (2011). Striving to respond to palliative care patients' pain at home: a puzzle for family caregivers. Oncol Nurs Forum.

[10] Mehta A, Chan LS, Cohen SR (2014). Flying blind: sources of distress for family caregivers of palliative cancer patients managing pain at home. J Psychosoc Oncol.

[11] Martín JM, Olano-Lizarraga M, Saracíbar-Razquin M (2016). The experience of family caregivers caring for a terminal patient at home: a research review. Int J Nurs Stud.

[12] Chi N, Demiris G (2017). Family caregivers' pain management in end-of-life care: a systematic review. Am J Hosp Palliat Care.

[13] Kelley M, Demiris G, Nguyen H, Oliver DP, Wittenberg-Lyles E (2013). Informal hospice caregiver pain management concerns: a qualitative study. Palliat Med.

[14] Oliver DP, Wittenberg-Lyles E, Washington K, Kruse RL, Albright DL, Baldwin PK, Boxer A, Demiris G (2013). Hospice caregivers' experiences with pain management: 'I'm not a doctor, and I don't know if I helped her go faster or slower'. J Pain Symptom Manage.

[15] Ferrell BR (2019). Family caregiving and cancer pain management. Anesth Analg.

[16] Green E, Ward S, Brierley W, Riley B, Sattar H, Harris T (2017). 'They shouldn't be coming to the ed, should they?': a descriptive service evaluation of why patients with palliative care needs present to the emergency department. Am J Hosp Palliat Care.

[17] Wallace EM, Cooney MC, Walsh J, Conroy M, Twomey F (2013). Why do palliative care patients present to the emergency department? Avoidable or unavoidable?. Am J Hosp Palliat Care.

[18] Oh TK, Jo YH, Choi JW (2018). Associated factors and costs of avoidable visits to the emergency department among cancer patients: 1-year experience in a tertiary care hospital in South Korea. Support Care Cancer.

[19] Deandrea S, Corli O, Consonni D, Villani W, Greco MT, Apolone G (2014). Prevalence of breakthrough cancer pain: a systematic review and a pooled analysis of published literature. J Pain Symptom Manage.

[20] Delgado-Guay MO, Kim YJ, Shin SH, Chisholm G, Williams J, Allo J, Bruera E (2015). Avoidable and unavoidable visits to the emergency department among patients with advanced cancer receiving outpatient palliative care. J Pain Symptom Manage.

[21] Phongtankuel V, Paustian S, Reid MC, Finley A, Martin A, Delfs J, Baughn R, Adelman RD (2017). Events leading to hospital-related disenrollment of home hospice patients: a study of primary caregivers' perspectives. J Palliat Med.

[22] Phongtankuel V, Scherban BA, Reid MC, Finley A, Martin A, Dennis J, Adelman RD (2016). Why do home hospice patients return to the hospital? A study of hospice provider perspectives. J Palliat Med.

[23] Kehl KA, Kowalkowski JA (2013). A systematic review of the prevalence of signs of impending death and symptoms in the last 2 weeks of life. Am J Hosp Palliat Care.

[24] Barbera L, Seow H, Howell D, Sutradhar R, Earle C, Liu Y, Stitt A, Husain A, Sussman J, Dudgeon D (2010). Symptom burden and performance status in a population-based cohort of ambulatory cancer patients. Cancer.

[25] Chi N, Demiris G, Pike KC, Washington K, Oliver DP (2018). Pain management concerns from the hospice family caregivers' perspective. Am J Hosp Palliat Care.

[26] Zadeh RS, Eshelman P, Setla J, Kennedy L, Hon E, Basara A (2018). Environmental design for end-of-life care: an integrative review on improving the quality of life and managing symptoms for patients in institutional settings. J Pain Symptom Manage.

[27] Littleton-Kearney MT, Grady PA (2018). The science of caregiving bringing voices together: summary of national institute of nursing research's 2017 summit. Nurs Outlook.

[28] von Ah D, Brown CG, Brown SJ, Bryant AL, Davies M, Dodd M, Ferrell B, Hammer M, Knobf MT, Knoop TJ, LoBiondo-Wood G, Mayer DK, Miaskowski C, Mitchell SA, Song L, Bruner DW, Wesmiller S, Cooley ME (2019). Research agenda of the oncology nursing society: 2019-2022. Oncol Nurs Forum.

[29] McGuire DB, Grant M, Park J (2012). Palliative care and end of life: the caregiver. Nurs Outlook.

[30] Rodríguez I, Herskovic V, Gerea C, Fuentes C, Rossel PO, Marques M, Campos M (2017). Understanding monitoring technologies for adults with pain: systematic literature review. J Med Internet Res.

[31] Silva EH, Lawler S, Langbecker D (2019). The effectiveness of mHealth for self-management in improving pain, psychological distress, fatigue, and sleep in cancer survivors: a systematic review. J Cancer Surviv.

[32] Allsop MJ, Taylor S, Mulvey MR, Bennett MI, Bewick BM (2015). Information and communication technology for managing pain in palliative care: a review of the literature. BMJ Support Palliat Care.

[33] Signorelli GR, Lehocki F, Fernández MM, O'Neill G, O'Connor D, Brennan L, Monteiro-Guerra F, Rivero-Rodriguez A, Hors-Fraile S, Munoz-Penas J, Bonjorn Dalmau M, Mota J, Oliveira RB, Mrinakova B, Putekova S, Muro N, Zambrana F, Garcia-Gomez JM (2019). A research roadmap: connected health as an enabler of cancer patient support. J Med Internet Res.

[34] Dickman Portz J, Ford K, Bekelman DB, Boxer RS, Kutner JS, Czaja S, Elsbernd K, Bull S (2020). 'We're taking something so human and trying to digitize': provider recommendations for mhealth in palliative care. J Palliat Med.

[35] Theile G, Klaas V, Tröster G, Guckenberger M (2017). Mhealth technologies for palliative care patients at the interface of in-patient to outpatient care: protocol of feasibility study aiming to early predict deterioration of patient's health status. JMIR Res Protoc.

[36] LeBaron V, Hayes J, Gordon K, Alam R, Homdee N, Martinez Y, Ogunjirin E, Thomas T, Jones R, Blackhall L, Lach J (2019). Leveraging smart health technology to empower patients and family caregivers in managing cancer pain: protocol for a feasibility study. JMIR Res Protoc.

[37] Etikan I (2016). Comparison of convenience sampling and purposive sampling. Am J Thery Appl Stat.

[38] Overview. NIH Common Fund.

[39] Blackhall LJ, Read P, Stukenborg G, Dillon P, Barclay J, Romano A, Harrison J (2016). CARE track for advanced cancer: impact and timing of an outpatient palliative care clinic. J Palliat Med.

[40] Cheng KK, Yeung RM (2013). Impact of mood disturbance, sleep disturbance, fatigue and pain among patients receiving cancer therapy. Eur J Cancer Care (Engl).

[41] Dodd MJ, Miaskowski C, Lee KA (2004). Occurrence of symptom clusters. J Natl Cancer Inst Monogr.

[42] Alam R, Dugan J, Homdee N, Gandhi N, Ghaemmaghami B, Meda H, Bankole A, Anderson M, Gong J, Smith-Jackson T, Lach J (2017). BESI: Reliable and Heterogeneous Sensing and Intervention for In-Home Health Applications. International Conference on Connected Health: Applications, Systems and Engineering Technologies.

[43] Bankole A, Anderson MS, Homdee N, Alam R, Lofton A, Fyffe N, Goins H, Newbold T, Smith-Jackson T, Lach J (2020). BESI: behavioral and environmental sensing and intervention for dementia caregiver empowerment-phases 1 and 2. Am J Alzheimers Dis Other Demen.

[44] Homdee N, Smith-Jackson T, Bankole A, Anderson MS, Lach J, Alam R, Hayes JA, Hamid T, Park J, Wolfe S, Goins H, Fyffe N, Newbold T (2019). Agitation monitoring and prevention system for dementia caregiver empowerment. Computer.

[45] Shiffman S, Stone AA, Hufford MR (2008). Ecological momentary assessment. Annu Rev Clin Psychol.

[46] Sandelowski M (2000). Whatever happened to qualitative description?. Res Nurs Health.

[47] Am I Rural? – Tool. Rural Health Information Hub.

[48] Oken MM, Creech RH, Tormey DC, Horton J, Davis TE, McFadden ET, Carbone PP (1982). Toxicity and response criteria of the eastern cooperative oncology group. Am J Clin Oncol.

[49] Schoppee TM, Dyal BW, Scarton L, Ezenwa MO, Singh P, Yao Y, Suarez ML, Wang ZJ, Molokie RE, Wilkie DJ (2019). Patients and caregivers rate the PainReportIt wireless internet-enabled tablet as a method for reporting pain during end-of-life cancer care. Cancer Nurs.

[50] Loh KP, Ramsdale E, Culakova E, Mendler JH, Liesveld JL, O'Dwyer KM, McHugh C, Gilles M, Lloyd T, Goodman M, Klepin HD, Mustian KM, Schnall R, Mohile SG (2018). Novel mhealth app to deliver geriatric assessment-driven interventions for older adults with cancer: pilot feasibility and usability study. JMIR Cancer.

[51] Creswell J, Poth C (2016). Qualitative Inquiry and Research Design: Choosing Among Five Approaches.

[52] Vasileiou K, Barnett J, Thorpe S, Young T (2018). Characterising and justifying sample size sufficiency in interview-based studies: systematic analysis of qualitative health research over a 15-year period. BMC Med Res Methodol.

[53] Børøsund E, Mirkovic J, Clark MM, Ehlers SL, Andrykowski MA, Bergland A, Westeng M, Solberg Nes L (2018). A stress management app intervention for cancer survivors: design, development, and usability testing. JMIR Form Res.

[54] Wolpin S, Halpenny B, Whitman G, McReynolds J, Stewart M, Lober W, Berry D (2015). Development and usability testing of a web-based cancer symptom and quality-of-life support intervention. Health Informatics J.

[55] Anderson K, Burford O, Emmerton L (2016). Mobile health apps to facilitate self-care: a qualitative study of user experiences. PLoS One.

[56] Morse JM (2016). Determining sample size. Qual Health Res.

[57] Langer SL, Ghosh N, Todd M, Randall AK, Romano JM, Bricker JB, Bolger N, Burns JW, Hagan RC, Porter LS (2020). Usability and acceptability of a smartphone app to assess partner communication, closeness, mood, and relationship satisfaction: mixed methods study. JMIR Form Res.

[58] Brick T, Mundie J, Weaver J, Fraleigh R, Oravecz Z (2020). Low-burden mobile monitoring, intervention, and real-time analysis using the wear-it framework: example and usability study. JMIR Form Res.

[59] Siemer L, Allouch SB, Pieterse M, Brusse-Keizer M, Sanderman R, Postel M (2020). Patients' user experience of a blended face-to-face and web-based smoking cessation treatment: qualitative study. JMIR Form Res.

[60] Sandelowski M (1995). Sample size in qualitative research. Res Nurs Health.

[61] Adam R, de Bruin M, Burton CD, Bond CM, Giatsi Clausen M, Murchie P (2018). What are the current challenges of managing cancer pain and could digital technologies help?. BMJ Support Palliat Care.

[62] Patel RA, Klasnja P, Hartzler A, Unruh KT, Pratt W (2012). Probing the benefits of real-time tracking during cancer care. AMIA Annu Symp Proc.

[63] (2017). Pain Management and the Opioid Epidemic. The National Academies Press.

[64] Paice JA (2018). Cancer pain management and the opioid crisis in America: how to preserve hard-earned gains in improving the quality of cancer pain management. Cancer.

[65] Paice JA, Von Roenn JH (2014). Under- or overtreatment of pain in the patient with cancer: how to achieve proper balance. J Clin Oncol.

[66] Joyce BT, Berman R, Lau DT (2014). Formal and informal support of family caregivers managing medications for patients who receive end-of-life care at home: a cross-sectional survey of caregivers. Palliat Med.

[67] Schumacher KL, Plano Clark VL, West CM, Dodd MJ, Rabow MW, Miaskowski C (2014). Pain medication management processes used by oncology outpatients and family caregivers part II: home and lifestyle contexts. J Pain Symptom Manage.

[68] Tabi K, Randhawa AS, Choi F, Mithani Z, Albers F, Schnieder M, Nikoo M, Vigo D, Jang K, Demlova R, Krausz M (2019). Mobile apps for medication management: review and analysis. JMIR Mhealth Uhealth.

[69] Han CJ, Chi N, Han S, Demiris G, Parker-Oliver D, Washington K, Clayton MF, Reblin M, Ellington L (2018). Communicating caregivers' challenges with cancer pain management: an analysis of home hospice visits. J Pain Symptom Manage.

[70] Schumacher KL, Plano Clark VL, West CM, Dodd MJ, Rabow MW, Miaskowski C (2014). Pain medication management processes used by oncology outpatients and family caregivers part I: health systems contexts. J Pain Symptom Manage.

[71] Gonella S, Sperlinga R, Sciannameo V, Dimonte V, Campagna S (2019). Characteristics of breakthrough pain and its impact on quality of life in terminally ill cancer patients. Integr Cancer Ther.

[72] Tagami K, Okizaki A, Miura T, Watanabe YS, Matsumoto Y, Morita T, Fujimori M, Kinoshita H (2018). Breakthrough cancer pain influences general activities and pain management: a comparison of patients with and without breakthrough cancer pain. J Palliat Med.

[73] Mercadante S, Marchetti P, Cuomo A, Caraceni A, Mediati RD, Vellucci R, Mammucari M, Natoli S, Lazzari M, Dauri M, Adile C, Airoldi M, Azzarello G, Bandera M, Blasi L, Cartenì G, Chiurazzi B, Costanzo BV, Degiovanni D, Fusco F, Guardamagna V, Iaffaioli V, Liguori S, Palermo L, Mameli S, Masedu F, Mattioli R, Mazzei T, Melotti RM, Menardo V, Miotti D, Moroso S, Pascoletti G, de Santis S, Orsetti R, Papa A, Ricci S, Scelzi E, Sofia M, Tonini G, Valle A, Aielli F, IOPS-MS Study Group (2018). Factors influencing the clinical presentation of breakthrough pain in cancer patients. Cancers (Basel).

[74] Finan PH, Goodin BR, Smith MT (2013). The association of sleep and pain: an update and a path forward. J Pain.

[75] Glover J, Dibble SL, Dodd MJ, Miaskowski C (1995). Mood states of oncology outpatients: does pain make a difference?. J Pain Symptom Manage.

[76] Amano K, Maeda I, Morita T, Okajima Y, Hama T, Aoyama M, Kizawa Y, Tsuneto S, Shima Y, Miyashita M (2016). Eating-related distress and need for nutritional support of families of advanced cancer patients: a nationwide survey of bereaved family members. J Cachexia Sarcopenia Muscle.

