# Supplementary material for: Understanding the Experience of Cancer Pain From the Perspective of Patients and Family Caregivers to Inform Design of an In-Home Smart Health System: Multimethod Approach
Source: JMIR Form Res. 2020 Aug 26;4(8):e20836. doi: 10.2196/20836 (PMC7481872; doi:10.2196/20836)
Supplement: Multimedia Appendix 1 [file formative_v4i8e20836_app1.docx]

A. Supplementary Materials

Participant Structured Interview Study Guide

**Part 1. Understanding the experience of cancer related pain at home.**

1. Have you/the patient had any cancer pain at home in the past week or so? If so, can you describe the experience from your perspective?

2. What has been the most difficult part of managing the pain at home?

3. What would help make managing the pain at home easier?

4. Our records indicate that you/the patient takes _______ for cancer pain [verify pain medication regimen with patient/caregiver].

- Do you/the patient have difficulty taking these medications? Do you have concerns about your/the pain medications?

**Part 2. Evaluating variables that may influence the experience of cancer pain at home.**Some things may make cancer pain better or worse. We are interested in knowing what those things may be from your perspective. I’d like to ask each of you your opinion. There is no right or wrong answer, and it is okay if you have different opinions. Please think back over the past few weeks or months.

Patient: For each item, I will ask you on a scale of 0 – 5 (0 = not at all, 5 = a great deal) how much do you think it makes your pain better or worse?

Caregiver: For each item, I’d like your opinion. On a scale of 0-5 (0 = not at all, 5 = a great deal) how much do you think it makes the patient’s pain better or worse?

| **Factor** | **Patient** | **Caregiver** | **Comment/Additional Context** |
| --- | --- | --- | --- |
| How long you sleep |  |  |  |
| How well you sleep |  |  |  |
| How happy or sad you feel |  |  |  |
| How much you are moving about; your activity level |  |  |  |
| How much you eat |  |  |  |
| How loud or noisy the environment is |  |  |  |
| How bright/light the room is |  |  |  |
| How warm or cold the room is |  |  |  |
| How dry or moist the air is |  |  |  |
| Taking your pain medication |  |  |  |
| How busy or hectic things are in the house |  |  |  |
| How physically close you are to each other |  |  |  |
| How emotionally close you feel to each other |  |  |  |
| Other (please list/describe) |  |  |  |

6. Are there any other items that you think make your pain or worse? [if so, add to chart or describe below; and rate on 0-5 scale]

**Part 3. Gathering feedback on the BESI-C system design.**

7. We would like to create a system that could help manage cancer pain at home. Your opinion about its design is very helpful. Part of the system could be monitors in different rooms of a person’s home. These monitors could measure things like how warm or bright a room is. Here are what the monitors could look like [show a picture or an actual sensor].

- What is your first impression of this monitor? What is the first word that comes to mind?
- Do you have any concerns about it being in your home?

8. Another part of the system could involve you both wearing a watch, such as this one: [show a picture or the actual smart watch]. This smart watch would measure things automatically (like how much you sleep or move about) but it would also ask you some questions during the day. For example, we would ask you to push the button if you are having pain and then answer some simple questions about the pain.

- What is your first impression of this smart watch? What is the first word that comes to mind?
- Would you be willing to answer questions on the watch? If so, how many times a day would be okay?
- What concerns do you have about wearing this type of watch?
- You could also answer questions on a smart phone or a tablet (like an iPad) like these [show a picture or actual devices] instead of the watch. Would you like that better? If so, why?
- What concerns do you have about answering questions on a smart phone or a tablet?

9. The final part of the system would be a laptop computer to collect and store information from the watch and room monitors. The laptop could be put in any room that is convenient for you, and you wouldn’t have to do anything with the laptop except keep it safe.

- Do you have any concerns about the laptop being in your home?

10. Do you have any other thoughts or ideas about what a home monitoring system for cancer pain should include?
